# A dual inhibition mechanism of herpesviral ICP47 arresting a conformationally thermostable TAP complex

**DOI:** 10.1038/srep36907

**Published:** 2016-11-15

**Authors:** Valentina Herbring, Anja Bäucker, Simon Trowitzsch, Robert Tampé

**Affiliations:** 1 Institute of Biochemistry, Biocenter, Goethe University Frankfurt, Max-von-Laue-Str. 9, 60438 Frankfurt a.M., Germany

## Abstract

As a centerpiece of antigen processing, the ATP-binding cassette transporter associated with antigen processing (TAP) became a main target for viral immune evasion. The herpesviral ICP47 inhibits TAP function, thereby suppressing an adaptive immune response. Here, we report on a thermostable ICP47-TAP complex, generated by fusion of different ICP47 fragments. These fusion complexes allowed us to determine the direction and positioning in the central cavity of TAP. ICP47-TAP fusion complexes are arrested in a stable conformation, as demonstrated by MHC I surface expression, melting temperature, and the mutual exclusion of herpesviral TAP inhibitors. We unveiled a conserved region next to the active domain of ICP47 as essential for the complete stabilization of the TAP complex. Binding of the active domain of ICP47 arrests TAP in an open inward facing conformation rendering the complex inaccessible for other viral factors. Based on our findings, we propose a dual interaction mechanism for ICP47. A *per se* destabilizing active domain inhibits the function of TAP, whereas a conserved C-terminal region additionally stabilizes the transporter. These new insights into the ICP47 inhibition mechanism can be applied for future structural analyses of the TAP complex.

Within the cellular process of antigen presentation via major histocompatibility complex class I (MHC I) molecules, the transporter associated with antigen processing TAP is responsible for antigen compartmentalization. As a centerpiece of the peptide loading complex, the heterodimeric ABC transporter TAP translocates proteasomal degradation products into the ER lumen, where they are loaded onto MHC I molecules. After editing and ER quality control, stable peptide-MHC I complexes traffic to the cell surface in order to present their antigenic cargo to cytotoxic T-lymphocytes. The antigen translocation complex is composed of two half-transporters, TAP1 and TAP2^1^,^2^, which can both be divided into three functional modules: an N-terminal transmembrane domain (TMD0), the central transmembrane domain (TMD), and the cytosolic nucleotide-binding domain (NBD)^3^,^4^. TMD and NBD form the coreTAP complex, which is connected by a short α-helix, named elbow helix, to the TMD0. The coreTAP complex is essential and sufficient for peptide binding and transport^3^, while the TMD0s are necessary for assembly of the peptide loading complex.

Viruses evolved elaborate strategies to inhibit MHC I antigen processing by interfering with TAP function^5^. For example, US6, the glycoprotein 6 of the cytomegalovirus unique short region, interacts with the ER-lumenal loops of TAP and prevents ATP binding at the cytosolic NBDs^6^,^7^,^8^,^9^,^10^. In herpes simplex viruses (HSV-1 and HSV-2), we find a distinct inhibition strategy, which also suppresses the MHC I surface presentation^11^. HSV infects mucosa and subsequently spreads via sensory neurons into ganglia, where it achieves a lifelong persistence^12^. After infection, cells start to synthesize infected cell polypeptides (ICPs). Five out of the more than fifty ICPs are the immediate early polypeptides ICP0, 4, 22, 27, and 47^13^,^14^, which regulate the expression of other ICPs or help to evade the host’s immune system. ICP47, also known as IE12, Vmw12, or IE5, binds to TAP from the cytosol (*K*_*D*_ = 50 nM) and arrests its function^15^,^16^,^17^,^18^,^19^. For ICP47 binding, both TAP subunits are required^16^,^17^, but the TMD0s are dispensable^20^. The active domain comprises residues 3-34 of ICP47, which displays the same inhibitory activity as the full-length protein^19^,^21^. The active domain undergoes a large conformational change from random-coil in solution into an α-helical structure in a lipid environment^22^. The structure of the active domain of ICP47 was determined by solution NMR^23^. It consists of two α-helices (residues 5-14 and 22-32) connected by a flexible linker. This helix-loop-helix conformation has been confirmed at phospholipid membranes by solid-state NMR^24^. A recent cryo-electron microscopy study mapped the helix-loop-helix conformation of ICP47 in the central cavity formed by the two TAP subunits^25^. However, the inhibition mechanism is still elusive and the EM model does not explain the biological function of the extended C-terminal region of ICP47 as to transport inhibition and its impact on the interaction with TAP.

Here, we established a fusion approach allowing to position the viral inhibitor ICP47 with a predefined orientation with regard to the heterodimeric TAP complex, thus creating an arrested ICP47-TAP complex. The otherwise dynamic TAP complex is trapped in its open inward-facing conformation, impeding the interaction with US6. This strategy allowed us to detect distinct behaviors of ICP47 fragments of the same length regarding each TAP subunit as well as to compare effects of ICP47 fragments of different lengths towards transport inhibition and thermal stabilization of the TAP complex. We demonstrate that the C-terminal extended region of ICP47 is necessary for complete stabilization of the TAP complex, whereas the N-terminal active domain is sufficient for TAP inhibition. Based on these data, we propose a dual inhibition mechanism by ICP47, which explains the high sequence conservation of the extended C-terminal region of ICP47.

## Results

### Design of ICP47-TAP fusion constructs

**Figure 1:**
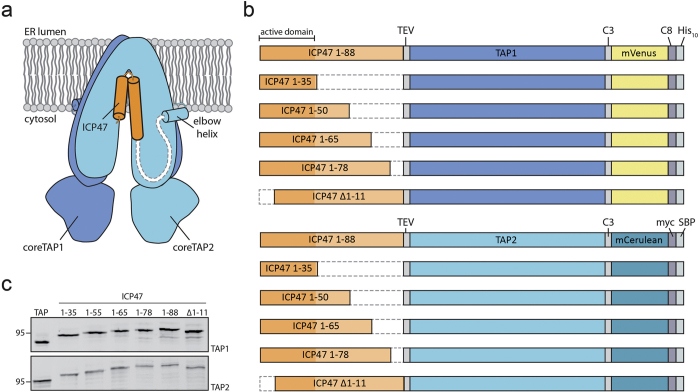
Design of ICP47-TAP fusion complexes. (**a**) Schematic representation of the ICP47-TAP fusion complexes. The ICP47 polypeptide is highlighted in orange and core TAP1 and TAP2 subunits in blue and light blue, respectively. (**b**) Six different ICP47 fragments (ICP47 1-35, 1-50, 1-65, 1-78, ∆1-11, and full length 1-88) were fused to the elbow helix of TAP1 or TAP2. For detection and purification, TAP1 and TAP2 were tagged with mVenus-C8-His_10_ or mCerulean-myc-SBP, respectively. Deleted regions are shown as dashed lines. TEV denotes a tobacco etch virus cleavage site and C3 a cleavage site for the human rhinovirus 3 C protease. The inactive ICP47 construct (ICP47 ∆1-11) served as a negative control. (**c**) Expression of TAP and ICP47-TAP fusions in HEK293T cells analyzed by SDS-PAGE and in-gel fluorescence.

We engineered ICP47-TAP fusions, which arrest the transporter by a defined high local concentration of ICP47 similarly to a ball-and-chain inactivation of voltage-gated ion channels^26^. In order to understand the function of the conserved C-terminal region of ICP47, we systematically truncated the polypeptide and fused it to the elbow helix of the TAP subunits (Figs 1a and S1). Five fusion constructs harboring the active domain of ICP47 (residues 1-35, 1-50, 1-65, 1-78, or 1-88) and one with an impaired active domain (residues 12-88) as negative control were generated with either TAP1 or TAP2 (Fig. 1b). To compare their expression levels and to allow purification of heterodimeric complexes, the TAP subunits were fused to the fluorescent proteins mVenus or mCerulean, followed by a C8-His_10_ tag and a myc-SBP tag, respectively. The N-terminal fusion of ICP47 to the TAP subunits and, in particular, the varying lengths of the ICP47 fragments did not affect the expression levels of the fusion constructs in human embryonic kidney cells (Fig. 1c).

### ICP47-TAP fusion complexes arrest the MHC I surface presentation

**Figure 2:**
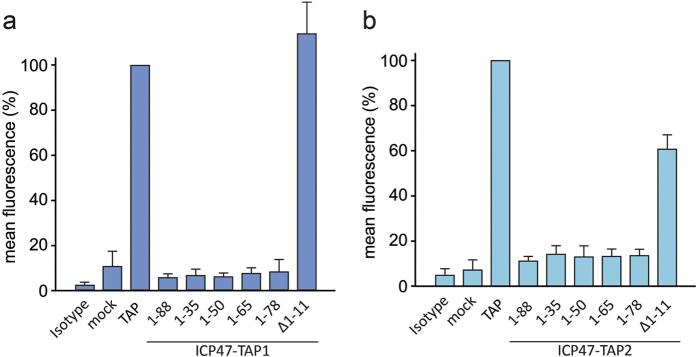
ICP47-TAP fusion complexes block MHC I surface expression. ICP47-TAP1 (**a**) and ICP47-TAP2 fusion variants (**b**) were expressed in the TAP-deficient cells BRE-169 (TAP1^−/−^) and STF1-169 (TAP2^−/−^), respectively. MHC I surface expression was monitored by flow cytometry using a PE-labeled MHC I-specific antibody. The mean PE fluorescence was calculated for transfected cells (mVenus positive). The mean (± SD) fluorescence of MHC I presented on the cell surface of TAP1- or TAP2-deficient cells transfected with TAP1 or TAP2 without ICP47 was normalized to 100%.

To test if peptide translocation is blocked by the ICP47 fusion constructs, we monitored MHC I surface expression by flow cytometry. The TAP-deficient human cell lines BRE-169 and STF1-169, isolated from patients lacking TAP1 or TAP2, respectively, were complemented with the missing subunit fused to different ICP47 fragments. The expression levels of the fusion constructs were examined by SDS-PAGE and in-gel fluorescence and the MHC I surface expression was quantified by flow cytometry. TAP-deficient cells transfected with a construct coding for mVenus only (mock control) display a background level of MHC I surface expression (Fig. 2). A similar background level was observed for all ICP47-TAP fusion constructs containing the active domain of ICP47. In contrast, the constructs harboring an N-terminally truncated ICP47 (Δ1-11) did not block MHC I surface expression in TAP-deficient cells. These changes in surface expression confirm that the active domain of ICP47 is necessary and sufficient for an effective interaction of the fused viral factor with TAP. Interestingly, the distance between the active domain of ICP47 (residues 3-34) and the elbow helix of each TAP subunit can vary between 1-62 amino acids without affecting the inhibitory function of the active domain, reflecting its high degree of flexibility including several interactions of ICP47 at the TAP1/2 interface.

### Asymmetric thermostability of ICP47-TAP complexes

**Figure 3:**
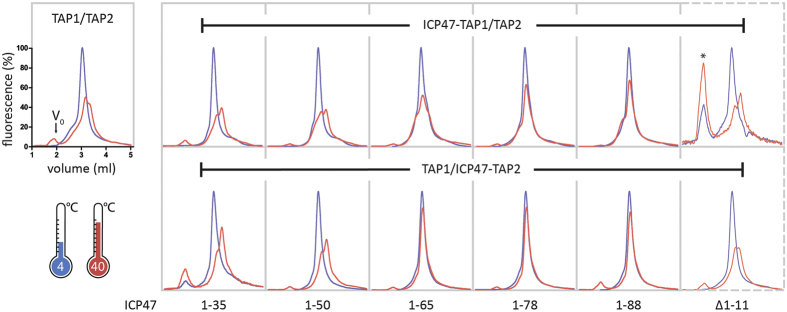
Fused ICP47 induces an asymmetric thermostability of TAP complexes. Purified TAP and ICP47-TAP complexes were analyzed by FSEC. Elution profiles of ICP47-TAP complexes were recorded by mVenus fluorescence after incubation at 4 °C (blue) and at 40 °C (red). FSEC profiles at 4 °C were normalized to 100%. The fusion constructs containing inactive ICP47(∆1-11) are displayed in dashed boxes. The void volume is indicated in the first panel. The peak denoted by an asterisk appears higher due to low purification yield.

Next, we co-expressed the ICP47-TAP fusion constructs with the complementary TAP subunit in human embryonic kidney cells. Assembly of stoichiometrically defined TAP1/2 complexes was analyzed by multicolor fluorescence-based size exclusion chromatography (MC-FSEC) after orthogonal purification via the His_10_- and the SBP-tag^27^. The TAP complex and all functionally arrested ICP47-TAP complexes elute as defined peaks (Fig. 3), corresponding to an apparent molecular weight of approximately 360 kDa. This estimated size indicates that the ICP47 fusions did not negatively affect TAP assembly. We then examined the thermostability of the ICP47-TAP complexes by MC-FSEC after incubation for one hour at 40 °C. Under these conditions, the TAP complex without fused ICP47 elutes as a double peak with reduced height, indicating that the complex is unstable at this temperature and eventually dissociates into its subunits (Fig. 3). As expected, ICP47-TAP complexes with an impaired active domain of ICP47 (∆1-11) do not show thermal stabilization. Surprisingly, TAP complexes fused to active ICP47 fragments spanning residues 1-35 and 1-50 are likewise unstable and elute as double peaks similar to TAP. The medium-length fragment of ICP47(1-65) fused to TAP1 also results in an unstable complex. Yet, a thermostable complex is assembled in case of a fusion to TAP2. TAP complexes containing ICP47 fragments 1-78 and 1-88 (full-length) are stable at 40 °C for 1 h. These findings suggest that the ICP47 fragments 1-35 and 1-50 fused to the elbow helix of either TAP1 or TAP2 did not allow an optimal positioning of the active domain in the binding cavity. The differences in thermal stabilization by ICP47(1-65) fusions demonstrate a directionality for optimal ICP47 binding that is dictated by the asymmetry of the TAP transporter.

### ICP47-TAP complexes are largely thermostabilized

**Figure 4:**
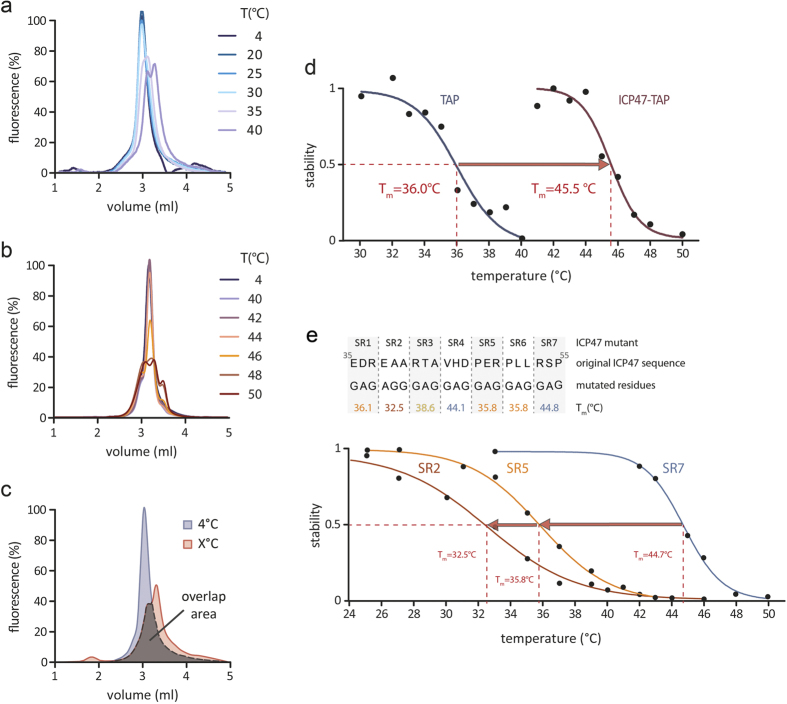
ICP47-TAP complexes display largely increased melting temperatures. Purified TAP (**a**) and TAP complex with ICP47(1-65) fused to TAP2 (**b**) were incubated at varying temperatures as indicated. FSEC profiles of mVenus fluorescence are shown. (**c**) Illustration of the overlap area calculation of TAP1/ICP47(1-50)-TAP2. (**d**) Melting curves were built using the overlap areas, here TAP1/2 and TAP1/ICP47(1-65)-TAP2 are shown exemplarily. The melting temperatures of all constructs are summarized in Table 1. (**e**) The stabilizing region was dissected into seven fragments (SR1-7) of three amino acids, which were replaced by glycines or alanines in order to link a change in melting temperature of TAP/ICP47 complexes to single residues of the stabilizing region. The calculated melting temperatures are depicted below the respective mutation. Melting curves of SR2, SR5, and SR7 are representatively shown.

We determined the melting temperature of ICP47-TAP complex exposed to increasing temperatures by analyzing MC-FSEC profiles. We assumed that the TAP subunits disintegrate at increasing temperatures, while the fluorescent reporter proteins stay intact. Thermostability profiles of heteromeric TAP and TAP1/ICP47-TAP2 fusion constructs are shown in Fig. 4a,b, respectively. By overlaying the MC-FSEC profiles with the reference peak at 4 °C, an overlap area can be calculated, which reflects the stability at defined temperatures (Fig. 4c). The melting curves of TAP1/2 and TAP1/ICP47(1-65)-TAP2 are exemplified in Fig. 4d. While detergent-solubilized TAP without fused ICP47 already dissociates at 36 °C, fusion of ICP47 raises the melting temperature of the TAP complexes to 47 °C (Table 1). Surprisingly, the shorter fragments 1-35 and 1-50 without any stabilizing effect on TAP1/2 in our thermostability assay increase the melting temperature by ~6 °C. The longer fragments 1-65, 1-78, and 1-88 (full-length), which stabilized TAP during a 1 h incubation at 40 °C, raised the melting temperature by ~10 °C.

For comparison, we also determined the melting temperature of TAP co-expressed with non-fused ICP47 variants, containing residues 1-35, 1-55, 1-73, and 1-88 (Table 1). Unexpectedly, the shortest fragment, ICP47(1-35), which has the same affinity as full-length ICP47 and is sufficient to block peptide binding and transport by TAP^19^,^21^, destabilizes the TAP complex. In contrast, ICP47(1-55), -(1-73), and -(1-88) raise the melting temperature to 45 °C, suggesting that residues 35-55 have an extra function in stabilizing the TAP complex. Taking into account that the 1-35 fragment fused to TAP2 increases the melting point by 5 °C, we hypothesize that the fusion *per se* helps to stabilize the TAP complex, circumventing the absence of the residues 35-55 and bringing the active domain in close proximity to the binding cavity.

To define the residues involved in the stabilization of TAP more precisely, we dissected the putative stabilizing region of ICP47 (residues 35-55) into seven segments of three residues (SR1-7). These triplets were exchanged for glycine-alanine-glycine, except for SR2, where alanine-glycine-glycine was used (Fig. 4e). We found the residues of SR2 to be the most critical for TAP stabilization. SR1, SR5, and SR6 lowered the melting temperature to 36 °C, SR3 to 38 °C. In contrast, SR4 and SR7 did not significantly affect the melting temperature of the TAP complex.

### ICP47-TAP fusion complexes are conformationally arrested

**Figure 5:**
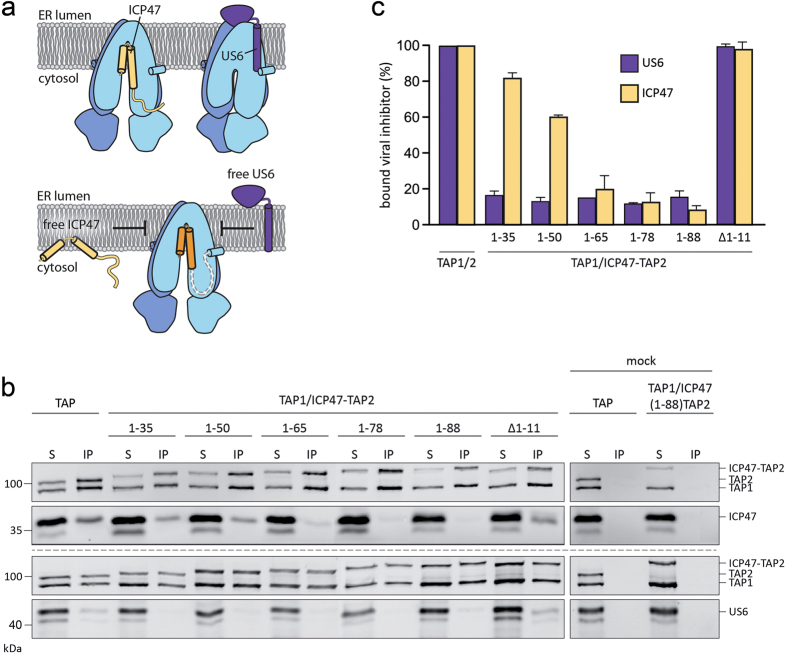
ICP47-TAP complexes arrest a conformation excluding viral proteins from binding. (**a**) ICP47 and US6 mutually exclude each other from binding to TAP (upper panel). The lower panel depicts the arrested conformation probed by binding of free ICP47 and US6. (**b**) In-gel fluorescence analysis of the viral proteins US6 and ICP47 co-expressed with ICP47-TAP complexes in HEK293T cells after co-immunoprecipitation via SPB- and C8-tags. Samples of solubilized complexes from whole cell extracts (S) and co-immunoprecipitated complexes (IP) are shown. (**c**) The co-precipitated viral factor was quantified in relation to the corresponding TAP complex lacking ICP47. Error bars represent standard deviations from three independent experiments.

In order to link the stabilizing effect to a physiological function, we probed the interaction of the ICP47-TAP fusion complexes with free viral factors. US6 and ICP47 interact with TAP from opposite subcellular compartments, the cytosol and the ER lumen, and exclude each other (Figs 5a and S2). To prove that the ICP47-TAP fusion complexes are arrested in a defined conformation, we co-expressed the ICP47-TAP fusion complexes and the free viral inhibitors. After co-immunoprecipitation, the interaction partners were quantified by SDS-PAGE and in-gel fluorescence (Fig. 5b). The TAP complex devoid of fused ICP47 presents maximal binding (100%) to the viral proteins (Fig. 5c). Notably, the interaction of non-fused ICP47 gradually decreases to background level with increasing size of the fused ICP47 fragments. The presence of the intact active domain is mandatory, since a truncation restores the amount of co-precipitated viral factor to 100%. Interestingly, we can distinguish between the most thermostable complexes (ICP47 1-65, 1-78, and 1-88) and unstable complexes (ICP47 1-35 and 1-50). In contrast, all ICP47-TAP fusion complexes do not interact with US6. Only a TAP fusion with an impaired active domain of ICP47(∆1-11) restores the interaction with US6, confirming that the entire active domain is required to arrest a defined conformation of TAP. Fused short fragments of ICP47 can be easily outcompeted by free full-length ICP47, although already a slight interaction with a fused short fragment causes the functional arrest. To conclude, an ICP47 fragment complying with all the requirements to generate a stably arrested TAP complex cannot be outcompeted by free viral factors.

## Discussion

This study reports on the identification of functionally arrested TAP complexes with high thermostability by fusing herpesviral ICP47 to TAP. Different lengths of ICP47 were chosen to map the optimal distance between the binding pocket and the N-terminal elbow helix of either TAP1 or TAP2. We show that the interaction of fused ICP47 with TAP inhibits antigen presentation via MHC I. Interestingly, the loss of MHC I surface expression only depends on the presence of the active domain and not on the length of the fused ICP47 fragments. All TAP complexes containing an intact active domain of ICP47 suppress MHC I surface expression. Contrarily, TAP complexes containing a fused inactive ICP47 restored the surface expression of MHC I. The ICP47-TAP fusion complexes revealed a preferred orientation for ICP47. The ICP47(1-65) fragment led to a stable complex only if fused to TAP2, highlighting an interesting asymmetry at the TAP1/TAP2 binding interface and suggesting a shorter distance of the C-terminus of the stabilization region to the elbow helix of TAP2 than of TAP1.

To define the thermostability more accurately, we determined the melting temperature of complexes with fused or freely bound ICP47 fragments. We showed that short fused fragments of ICP47 (residues 1-35 or 1-50) did not fully stabilize the TAP complex in thermostability assays. Only ICP47 fragments longer than residues 1-50 raised the melting temperature to the full extent and led to a completely stabilized complex. Furthermore, free viral fragments in complex with TAP revealed ICP47(1-55) as the shortest fragment that stabilizes TAP, while shorter fragments destabilized the complex. This destabilization is also reflected by MHC I surface expression, where only free fragments of at least residues 1-55 prevent MHC I surface expression to its full extent (Fig. S3).

While the affinity constants of fully inhibiting ICP47 fragments are similar, they presumably modulate the structure of the TAP transporter in different ways^19^. Surprisingly, the active domain alone, which was used to study the TAP function^3^,^20^,^24^, decreased thermostability, while the fused active domain alone led to a slight increase in stability. ICP47 interacts via a large interface with TAP, involving most of the residues of the active domain of ICP47^21^. Therefore, we postulate that a fused active domain, which does not reach the optimal position inside the binding cavity, can still impair the function of the TAP complex. Consequently, non-ideally positioned fused ICP47 can be outcompeted by free full-length ICP47. The functional arrest is demonstrated by the inability of an ICP47-TAP complex to bind free US6.

**Figure 6:**
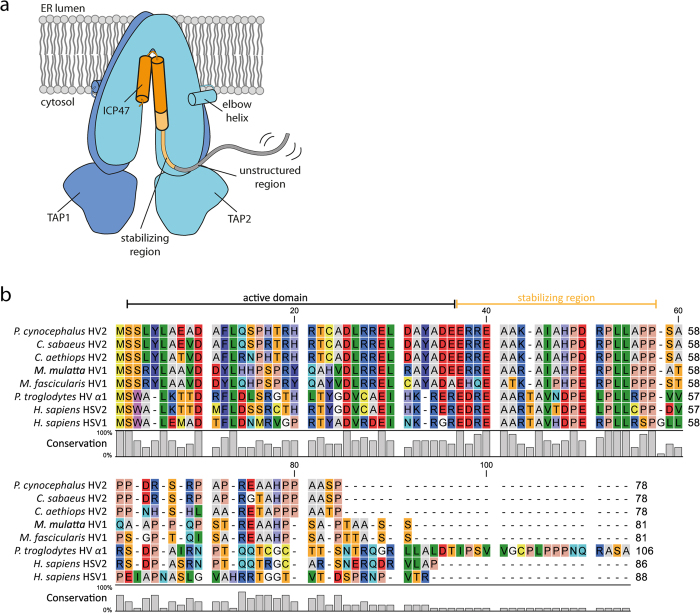
ICP47 proteins encoded by different herpes viruses share conserved sequences. (**a**) Schematic representation of the TAP and ICP47 interaction. The stabilizing region of ICP47 (35-55) is highlighted in yellow, the unstructured region depicted in grey. (**b**) When comparing different ICP47s from the herpesviral clade with human ICP47, a high conservation can be found for the residues 1-55. The PxxPLLxPP motif, corresponding to the residues 50-55 of human HSV2, can be found in all ICP47 proteins, except for human HSV1. Alignments were created with CLC Sequence Viewer 7.6.1. and skylign (skylign.org). NCBI accession.version: *P. c.* HV2, YP_443919.1; *C. s.* HV2, AAW78015.1; *C. a.* HV2, YP_164515.1; *M. m.* HV1, NP_851932.1; M*. f.* HV1, AIA09566.1; *P. t.* HV *α* 1, YP_009011060.1; *H. s* HSV1, AMB65879.1; *H. s* HSV2, AMB66178.1.

The active domain (residues 3-34) was defined to be the shortest region with the same inhibitory activity as full-length ICP47^19^,^21^. Despite a high conservation of residues following the active domain (residues 35-55), no function could be assigned to that region^19^,^21^. By comparing ICP47 sequences derived from HSV1 and HSV2, residues 1-55 are highly conserved with a sequence identity of 67%, while residues 56-88 show no significant conservation (Fig. 6b). Furthermore, a comparison of different ICP47s from the herpesviral clade reveals a high number of charged residues, a short alanine-rich sequence, and a proline-rich PxxPLLxPP motif in the C-terminal region (Fig. 6b). Since proline-rich motifs are known to be involved in protein-protein interactions^28^, we hypothesize that such a motif has also evolved in the ICP47-TAP interaction to lock the transporter in a peptide-repellent conformation. By exchanging amino acid triplets in the stabilizing region, we could prove the importance of both the alanine- and the proline-rich sequences. Furthermore, we could demonstrate that the most conserved residues are the most critical for a complete stabilization of the TAP complex.

In summary, our findings reveal a dual inhibition mechanism of ICP47. While the active domain of ICP47 is wedged at the TAP1/2 interface and arrests the complex in an open-inward facing conformation, the highly conserved C-terminal region stabilizes the ICP47-TAP interaction and generates a thermostabilized TAP complex. Due to the recent progress in using stabilizing interaction partners for structure determination by X-ray crystallography and single particle cryo-electron microscopy, these stabilized TAP complexes will be an interesting basis to substantiate the ICP47/TAP inhibition mechanism to a single-residue level.

## Materials and Methods

### Cloning and construct design

All constructs are based on *de novo* synthesized coreTAP1- (UniProt knowledgebase number Q03518; residues 224-808) and coreTAP2-sequences (UniProt knowledgebase number Q59H06; residues 124-704), where all cysteines except C213_TAP2_ are replaced. This single intrinsic cysteine is required to maintain a peptide-receptive conformation by allocating the peptide in the correct orientation into the binding pocket^29^,^30^. All cloning steps were based on PCR amplification by *Pfu* DNA polymerase and FX-cloning^31^. All constructs were verified by DNA sequencing. ICP47 fragments of variable length (residues 1-35, 1-50, 1-65, 1-78, 1-88, or 12-88) were N-terminally fused to either coreTAP1 or coreTAP2 and transferred into the mammalian FX-expression vectors pcDXc3YCH (ICP47-TAP1 and ICP47-TAP2, for C-terminal mVenus-C8-His_10_ tag) and pcDXc3CMS (ICP47-TAP2, for C-terminal mCerulean-myc-SBP tag)^32^. The ICP47-TAP fusion constructs for expression in human cells and subsequent orthogonal purification contained a tobacco etch virus (TEV) cleavage site between the ICP47 and coreTAP1 or coreTAP2. For flow cytometry, mVenus-tagged ICP47-TAP2 constructs were used (pcDXc3YCH vector). Coding sequences for non-fused ICP47 fragments (residues 1-35, 1-55, 1-73, and 1-88) and US6 were cloned into pcDXc3YMH (for C-terminal mVenus-myc-His_10_ tag) to avoid tag-interference in the co-immunoprecipitation (IP) procedure.

### Cell lines and cell culture

BRE-169 and STF1-169 cell lines were kindly provided by H. de la Salle, Strasbourg, France^33^,^34^. HEK293T, BRE-169, and STF1-169 cells were cultured in DMEM (Gibco) supplemented with 10% fetal calf serum (FCS, Gibco). FreeStyle™ 293-F cells were cultured on plates in DMEM (Gibco) supplemented with 10% FCS and in FreeStyle™ 293 Expression Medium (Gibco) in suspension. Adherent cell lines were cultured at 37 °C and 8% CO_2_. FreeStyle 293-F cells in suspension were cultured at 37 °C, 8.1% CO_2_ and 125 rpm.

### Transfection of HEK293T cells

5 × 10^5^ HEK293T cells per well were seeded in 6-well plates in DMEM supplemented with 10% FCS. After 24 h, cells were transfected using polyethyleneimine (PEI). 4 μg DNA and 15 μl PEI (1 mg/ml) were dissolved separately in 200 μl serum-free DMEM, subsequently mixed, and incubated for 30 min at room temperature. The transfection solution was added dropwise to the wells. The cells were harvested 24 h after transfection. For large scale, three days prior to transfection, 2 × 10^6^ HEK293T cells were seeded in 15 cm dishes in 15 ml DMEM supplemented with 10% FCS. 30 μg of DNA (equimolar ratio of the two subunits) and 90 μl PEI (1 mg/ml) were dissolved separately in 4.2 ml serum-free DMEM, the solutions were mixed and incubated for 30 min at room temperature. The transfection solution was added dropwise to the dishes. The cells were harvested 24 h after transfection.

### Transfection of FreeStyle 293-F cells

1.5 × 10^8^ cells were seeded in 300 ml FreeStyle™ 293 expression medium. After 24 h, cells were transfected using PEI. 300 μg DNA and 1.2 ml PEI(1 mg/ml) were dissolved separately in 10 ml Opti-MEM I (1X) + GlutaMAX™ reduced serum medium (Gibco). The solutions were mixed and incubated for 30 min at room temperature and then added to the dishes. The cells were harvested 65 h after transfection.

### Transfection of TAP-deficient cells

2.5 × 10^5^ BRE-169 or STF1-169 cells per well were seeded in 6-well plates in DMEM supplemented with 10% FCS. After 24 h, cells were transfected using Xtreme-GENE HP (Roche). 2 μg DNA and 6 μl Xtreme-GENE HP were dissolved separately in 200 μl serum-free DMEM, subsequently mixed, and incubated for 30 min at room temperature. The transfection solution was added dropwise to the wells. The cells were harvested 48 h after transfection.

### In-gel fluorescence

The transfected cells of one 6-well plate were harvested and centrifuged for 4 min at 1,000 × g. Cells were lysed in 70 μl Pierce RIPA buffer (Thermo Scientific) supplemented with 1% benzonase (Novagen, EMD Chemicals) and subsequently incubated for 1 h at room temperature. Sodium dodecyl sulfate (SDS)-buffer was added to a final concentration of 62.5 mM Tris/HCl, pH 6.8, 100 mM β-mercaptoethanol, 2% SDS, 0.02% bromophenol blue and 10% glycerol. 1.2 × 10^5^ cells were loaded on a 10% SDS-PA-gel (Laemmli) and run for 1 h at 125 V. In-gel fluorescence was detected using an ImageQuant LAS 4000 (GE Healthcare Life Sciences) at emission and excitation wavelengths (λ) of 460 and 515 nm, respectively, to detect fluorescence of mCerulean and mVenus, or at 520 and 575 nm, respectively, to detect mVenus fluorescence only. For visualization of the marker bands, the gel was analyzed at emission and excitation wavelengths (λ) of 630 and 670 nm, respectively.

### Flow cytometry

MHC I surface expression was analyzed by flow cytometry. TAP1-deficient BRE-169 cells and TAP2-deficient STF1-169 cells were complemented each with the missing subunit fused to ICP47 fragments. After harvesting the cells, all further steps were carried out on ice. The cells were washed in FACS-buffer (2% FCS in PBS, ice-cold) and centrifuged 3 min at 300 × g and 4 °C. After discarding the supernatant, cells were washed twice in FACS-buffer. Cells were then blocked 20 min by BSA in FACS-buffer. After two more washing steps, the cells were stained 1 h with 125 ng PE anti-human HLA-A,B,C (clone W6/32; BioLegend) in 100 μl FACS-buffer. The non-bound antibody was removed by washing with FACS-buffer. The cells were fixed with 0.25% formaldehyde in FACS-buffer and stored at 8 °C or used immediately for cytometry. Data were recorded by an Attune (Invitrogen) flow cytometer and processed using FlowJo 8.8.7 software (TreeStar, Inc.).

### Purification of ICP47-TAP complexes

For the orthogonal purification of TAP complexes, all steps were carried out on ice and all buffers were adjusted to pH 7.4. The transfected and harvested cells of six 15 cm dishes were solubilized for 2 h in buffer 1 (20 mM HEPES/NaOH, 200 mM NaCl, 50 mM KCl and 15% (v/v) glycerol) supplemented with 10 mM imidazole, 1 × PI-mix HP (Serva), and 2% (w/v) glyco-diosgenin (GDN, Anatrace). The samples were centrifuged 30 min at 120,000 × g and 4 °C. The proteins were bound to 300 μl Ni^2+^-resin (Ni^2+^-Sepharose 6 Fast Flow, GE Healthcare) for 2 h. The beads were washed twice 15 min with buffer 1 supplemented with 10 mM imidazole and 0.05% GDN. To elute the proteins, the beads were incubated 30 min in buffer 1 supplemented with 200 mM imidazole and 0.05% GDN. The eluate was incubated for 3 h with 200 μl streptavidin resin (High Capacity Streptavidin Agarose Resin, Thermo Scientific). The beads were washed twice for 15 min with buffer 1 supplemented with 0.05% GDN. Subsequently, bound proteins were eluted for 45 min in buffer 1 supplemented with 2.5 mM biotin and 0.05% GDN. The eluate was frozen in liquid nitrogen and stored at −80 °C.

### MC-FSEC and thermostability analyses

The monodispersity of the purified TAP complexes was analyzed by multicolor fluorescence-based size exclusion chromatography (MC-FSEC)^27^. The fluorescent proteins fused to TAP subunits (mVenus on TAP1 and mCerulean on TAP2) or ICP47 were detected by an Agilent 1200 series high-performance liquid chromatography (HPLC) system using a Shodex semi-micro KW404-4 F (4.66 × 300 mm) column. The running buffer was composed of 20 mM HEPES/NaOH pH 7.4, 200 mM NaCl, 50 mM KCl, 5% glycerol, and 0.05% GDN. For the MC-FSEC analysis, 60 μl of the purified TAP complexes were filtered through 0.22 μm SpinX centrifuge filter (Costar). 50 μl of each sample were then injected by an autosampler. To determine the thermostability of the ICP47-TAP complexes, 60 μl of the protein solution were incubated for 1 h at 40 °C, then treated as described above, and compared by MC-FSEC with a sample stored for 1 h on ice. The melting points of the protein complexes were determined by incubating samples five min at 25–50 °C. Elution profiles were overlaid and the overlap area was calculated with ImageJ 1.48 v (Fig. 4c). The area of the profiles at 4 °C was normalized to 100%. Curve fit was calculated with GraphPad Prism 5 using the nonlinear regression “EC50 shift fit”.

### Co-immunoprecipitation

To analyze the binding behavior of the ICP47-TAP complexes to free ICP47 and US6 (C-terminal mVenus-tag), HEK293T cells were transfected in 15 cm dishes (1 dish per construct, 16 × 10^6^ cells) with an excess of free expressed viral factor. A molar ratio of 1:1:5 (TAP1:TAP2:viral factor) was applied for transfection using 30 μg total DNA and 90  μl PEI (1 mg/ml) per dish. The cells were harvested and solubilized for 1 h in 1 ml of buffer 1 supplemented with 1 × PI-mix HP (Serva) and 2% digitonin (Merck Millipore). All steps were carried out on ice. An aliquot of every step was stored in SDS buffer (50 mM Tris/HCl, pH 7.5, 10% glycerol, 2% SDS, 0.7% β-mercaptoethanol). The samples were centrifuged for 30 min at 120,000 × g and 4 °C. Afterwards, the supernatant was incubated for 1 h with 50 μl prewashed streptavidin agarose beads (High Capacity Streptavidin Agarose Resin, Thermo Scientific). For washing, the beads were incubated for 15 min in buffer 1 supplemented with 0.1% digitonin. Bound proteins were eluted for 15 min in buffer 1 supplemented with 2.5 mM biotin and 0.1% digitonin. 8 μg of anti-C8 antibody^35^ were bound to 50 μl of sheep anti-mouse IgG Dynabeads (Novex™, Life-Technologies) for 1 h and washed twice with buffer 1 supplemented with 0.1% digitonin. The eluted proteins were added to the antibody-coated Dynabeads and incubated for 90 min. The beads were washed three times for 5 min in buffer 1 supplemented with 0.1% digitonin and then eluted for 30 min at 37 °C in SDS buffer.

## Additional Information

**How to cite this article**: Herbring, V. *et al.* A dual inhibition mechanism of herpesviral ICP47 arresting a conformationally thermostable TAP complex. *Sci. Rep.* **6**, 36907; doi: 10.1038/srep36907 (2016).

**Publisher’s note:** Springer Nature remains neutral with regard to jurisdictional claims in published maps and institutional affiliations.

## Supplementary Material

Supplementary Information

## Figures and Tables

**Table 1:** Stabilization of ICP47-TAP complexes.

construct	T_m_ (°C)	
TAP1/TAP2	36.0	
TAP1/ICP47(1-35)TAP2	41.2	fused ICP47
TAP1/ICP47(1-50)TAP2	43.4	
TAP1/ICP47(1-65)TAP2	45.5	
TAP1/ICP47(1-78)TAP2	45.6	
TAP1/ICP47(1-88)TAP2	47.0	
TAP1/TAP2/ICP47(1-35)	28.2	free ICP47
TAP1/TAP2/ICP47(1-55)	44.6	
TAP1/TAP2/ICP47(1-73)	45.1	
TAP1/TAP2/ICP47(1-88)	45.7	

## References

[b1] Trowsdale J. *et al.* Sequences encoded in the class II region of the MHC related to the ‘ABC’ superfamily of transporters. Nature 348, 741–744 (1990). 2259383 10.1038/348741a0

[b2] Deverson E. V. *et al.* MHC class II region encoding proteins related to the multidrug resistance family of transmembrane transporters. Nature 348, 738–741 (1990). 1979660 10.1038/348738a0

[b3] Koch J., Guntrum R., Heintke S., Kyritsis C. & Tampé R. Functional dissection of the transmembrane domains of the transporter associated with antigen processing (TAP). J Biol Chem 279, 10142–10147 (2004). 14679198 10.1074/jbc.M312816200

[b4] Koch J. & Tampé R. The macromolecular peptide-loading complex in MHC class I-dependent antigen presentation. Cell Mol Life Sci 63, 653–662 (2006). 16465444 10.1007/s00018-005-5462-z PMC11136332

[b5] Mayerhofer P. U. & Tampé R. Antigen translocation machineries in adaptive immunity and viral immune evasion. J Mol Biol 427, 1102–1118 (2015). 25224907 10.1016/j.jmb.2014.09.006

[b6] Lehner P. J., Karttunen J. T., Wilkinson G. W. & Cresswell P. The human cytomegalovirus US6 glycoprotein inhibits transporter associated with antigen processing-dependent peptide translocation. Proc Natl Acad Sci USA 94, 6904–6909 (1997). 9192664 10.1073/pnas.94.13.6904 PMC21257

[b7] Hengel H. *et al.* A viral ER-resident glycoprotein inactivates the MHC-encoded peptide transporter. Immunity 6, 623–632 (1997). 9175840 10.1016/s1074-7613(00)80350-7

[b8] Ahn K. *et al.* The ER-luminal domain of the HCMV glycoprotein US6 inhibits peptide translocation by TAP. Immunity 6, 613–621 (1997). 9175839 10.1016/s1074-7613(00)80349-0

[b9] Kyritsis C. *et al.* Molecular mechanism and structural aspects of transporter associated with antigen processing inhibition by the cytomegalovirus protein US6. J Biol Chem 276, 48031–48039 (2001). 11606590 10.1074/jbc.M108528200

[b10] Hewitt E. W., Gupta S. S. & Lehner P. J. The human cytomegalovirus gene product US6 inhibits ATP binding by TAP. EMBO J 20, 387–396 (2001). 11157746 10.1093/emboj/20.3.387 PMC133477

[b11] York I. A. *et al.* A cytosolic herpes simplex virus protein inhibits antigen presentation to CD8+ T lymphocytes. Cell 77, 525–535 (1994). 8187174 10.1016/0092-8674(94)90215-1

[b12] Whitley R. J., Kimberlin D. W. & Roizman B. Herpes simplex viruses. Clin Infect Dis 26, 541–553; quiz 554–555 (1998). 9524821 10.1086/514600

[b13] Honess R. W. & Roizman B. Proteins specified by herpes simplex virus. XI. Identification and relative molar rates of synthesis of structural and nonstructural herpes virus polypeptides in the infected cell. J Virol 12, 1347–1365 (1973). 4357511 10.1128/jvi.12.6.1347-1365.1973 PMC356776

[b14] Watson R. J., Preston C. M. & Clements J. B. Separation and characterization of herpes-simplex virus type-1 immediate-early messenger-RNAs. J. Virol. 31, 42–52 (1979). 228058 10.1128/jvi.31.1.42-52.1979 PMC353420

[b15] Früh K. *et al.* A viral inhibitor of peptide transporters for antigen presentation. Nature 375, 415–418 (1995). 7760936 10.1038/375415a0

[b16] Hill A. *et al.* Herpes simplex virus turns off the TAP to evade host immunity. Nature 375, 411–415 (1995). 7760935 10.1038/375411a0

[b17] Ahn K. *et al.* Molecular mechanism and species specificity of TAP inhibition by herpes simplex virus protein ICP47. EMBO J 15, 3247–3255 (1996). 8670825 PMC451885

[b18] Tomazin R. *et al.* Stable binding of the herpes simplex virus ICP47 protein to the peptide binding site of TAP. EMBO J 15, 3256–3266 (1996). 8670826 PMC451888

[b19] Neumann L., Kraas W., Uebel S., Jung G. & Tampé R. The active domain of the herpes simplex virus protein ICP47: A potent inhibitor of the transporter associated with antigen processing (TAP). J Mol Biol 272, 484–492 (1997). 9325106 10.1006/jmbi.1997.1282

[b20] Eggensperger S., Fisette O., Parcej D., Schafer L. V. & Tampé R. An annular lipid belt is essential for allosteric coupling and viral inhibition of the antigen translocation complex TAP (transporter associated with antigen processing). J Biol Chem 289, 33098–33108 (2014). 25305015 10.1074/jbc.M114.592832 PMC4246070

[b21] Galocha B. *et al.* The active site of ICP47, a herpes simplex virus-encoded inhibitor of the major histocompatibility complex (MHC)-encoded peptide transporter associated with antigen processing (TAP), maps to the NH_2_-terminal 35 residues. J Exp Med 185, 1565–1572 (1997). 9151894 10.1084/jem.185.9.1565 PMC2196299

[b22] Beinert D., Neumann L., Uebel S. & Tampé R. Structure of the viral TAP-inhibitor ICP47 induced by membrane association. Biochemistry 36, 4694–4700 (1997). 9109681 10.1021/bi962940v

[b23] Pfänder R. *et al.* Structure of the active domain of the herpes simplex virus protein ICP47 in water/sodium dodecyl sulfate solution determined by nuclear magnetic resonance spectroscopy. Biochemistry 38, 13692–13698 (1999). 10521276 10.1021/bi9909647

[b24] Aisenbrey C. *et al.* Structure and dynamics of membrane-associated ICP47, a viral inhibitor of the MHC I antigen-processing machinery. J Biol Chem 281, 30365–30372 (2006). 16835230 10.1074/jbc.M603000200

[b25] Oldham M. L. *et al.* A mechanism of viral immune evasion revealed by cryo-EM analysis of the TAP transporter. Nature 529, 537–540 (2016). 26789246 10.1038/nature16506 PMC4848044

[b26] Armstrong C. M. & Bezanilla F. Inactivation of the sodium channel. II. Gating current experiments. J Gen Physiol 70, 567–590 (1977). 591912 10.1085/jgp.70.5.567 PMC2228472

[b27] Parcej D., Guntrum R., Schmidt S., Hinz A. & Tampé R. Multicolour fluorescence-detection size-exclusion chromatography for structural genomics of membrane multiprotein complexes. PLoS One 8, e67112 (2013). 23825631 10.1371/journal.pone.0067112 PMC3692423

[b28] Kay B. K., Williamson M. P. & Sudol P. The importance of being proline: the interaction of proline-rich motifs in signaling proteins with their cognate domains. Faseb Journal 14, 231–241 (2000). 10657980

[b29] Heintke S. *et al.* Functional cysteine-less subunits of the transporter associated with antigen processing (TAP1 and TAP2) by de novo gene assembly. FEBS Lett 533, 42–46 (2003). 12505156 10.1016/s0014-5793(02)03746-8

[b30] Baldauf C., Schrodt S., Herget M., Koch J. & Tampé R. Single residue within the antigen translocation complex TAP controls the epitope repertoire by stabilizing a receptive conformation. Proc Natl Acad Sci USA 107, 9135–9140 (2010). 20439763 10.1073/pnas.1001308107 PMC2889111

[b31] Geertsma E. R. & Dutzler R. A versatile and efficient high-throughput cloning tool for structural biology. Biochemistry 50, 3272–3278 (2011). 21410291 10.1021/bi200178z

[b32] Hinz A. *et al.* Assembly and function of the major histocompatibility complex (MHC) I peptide-loading complex are conserved across higher vertebrates. J Biol Chem 289, 33109–33117 (2014). 25320083 10.1074/jbc.M114.609263 PMC4246071

[b33] de la Salle H. *et al.* Homozygous human TAP peptide transporter mutation in HLA class I deficiency. Science 265, 237–241 (1994). 7517574 10.1126/science.7517574

[b34] de la Salle H. *et al.* HLA class I deficiencies due to mutations in subunit 1 of the peptide transporter TAP1. J Clin Invest 103, R9–R13 (1999). 10074495 10.1172/JCI5687 PMC408129

[b35] Abacioglu Y. H. *et al.* Epitope mapping and topology of baculovirus-expressed HIV-1 gp160 determined with a panel of murine monoclonal antibodies. AIDS Res Hum Retroviruses 10, 371–381 (1994). 8068416 10.1089/aid.1994.10.371

