# Supplementary material for: A dual inhibition mechanism of herpesviral ICP47 arresting a conformationally thermostable TAP complex
Source: Sci Rep. 2016 Nov 15;6:36907. doi: 10.1038/srep36907 (PMC5109273; doi:10.1038/srep36907)
Supplement: Supplementary Information [file srep36907-s1.pdf]

A dual inhibition mechanism of herpesviral ICP47 arresting a conformationally thermostable TAP complex

Valentina Herbring, Anja Bäucker, Simon Trowitzsch, and Robert Tampé[1]*

[1]Institute of Biochemistry, Biocenter, Goethe University Frankfurt, Max-von-Laue-Str. 9, 60438 Frankfurt a.M., Germany.

*For correspondence: tampe@em.uni-frankfurt.de

Supplementary Figures

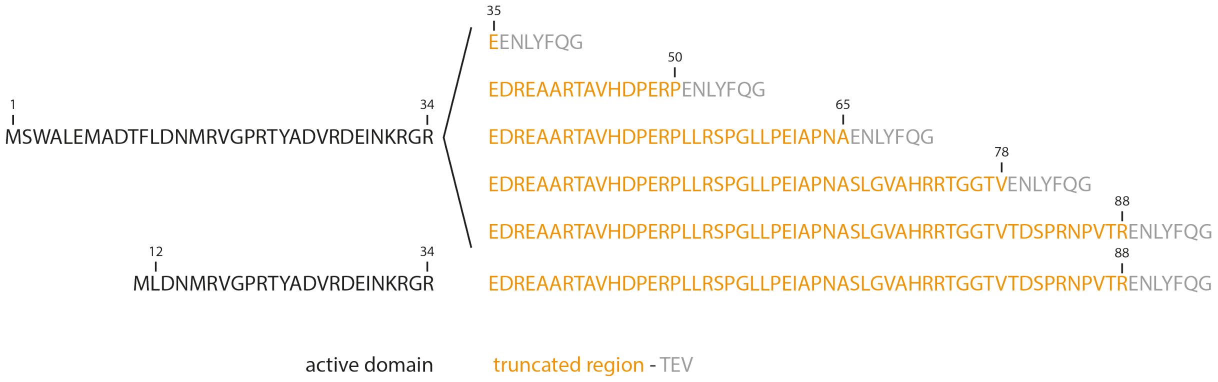

Figure S1. ICP47 fragments used for ICP47-TAP fusion. The active domain of ICP47 (residues 1-34) is depicted in black, the C-terminal region of ICP47 in orange and the TEV cleavage site in grey. The truncated regions serve as natural linkers between the active domain and the TAP subunits.

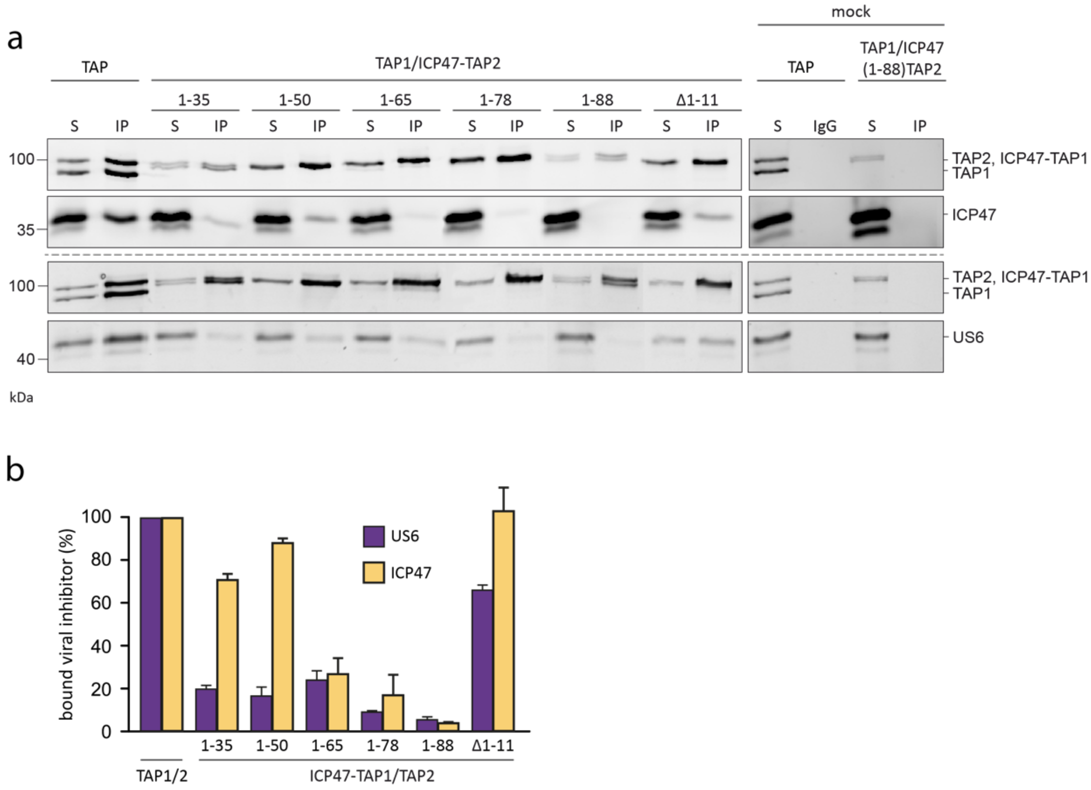

Figure S2. ICP47-TAP complexes arrest a conformation excluding viral proteins from binding. (**a**) In-gel fluorescence analysis of the viral proteins US6 and ICP47 co-expressed with ICP47-TAP1/TAP2 complexes in HEK293T cells after co-immunoprecipitation via SPB- and C8-tags. Samples of solubilized complexes from whole cell extracts (S) and co-immunoprecipitated complexes (IP) are shown. (**b**) The co-precipitated viral factor was quantified in relation to the corresponding TAP complex lacking ICP47. Error bars represent standard deviations from three independent experiments.

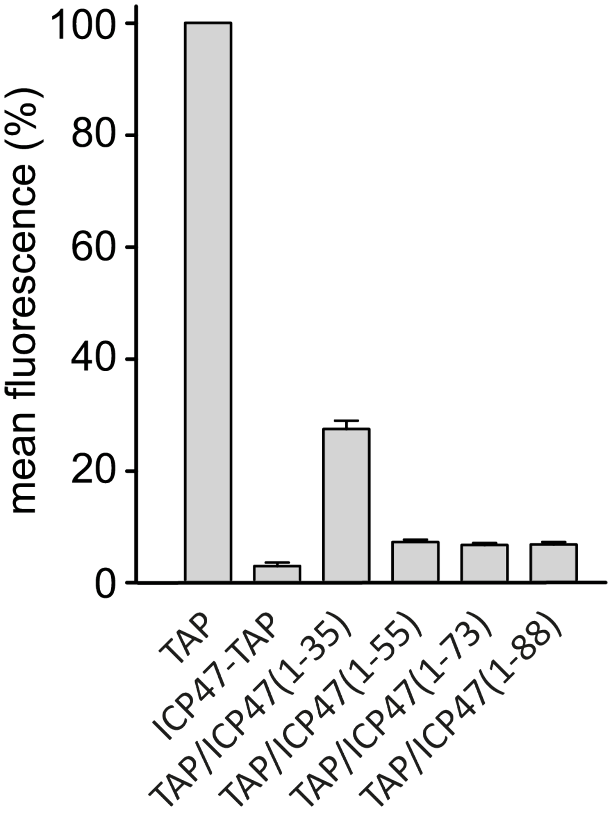

Figure S3. Free ICP47 fragments arrest TAP and block MHC I surface expression. Free ICP47 fragments 1-35, 1-55, 1-73, and 1-88 were co-expressed with TAP in the TAP-deficient cells BRE-169 ($TAP1^{-/-}$). MHC I surface expression was monitored by flow cytometry using a PE-labeled MHC I-specific antibody. The mean PE fluorescence was calculated for transfected cells (mVenus positive). The mean fluorescence of MHC I presented on the cell surface of TAP1-deficient cells transfected with TAP1 was normalized to 100%.
